# Design and Characterisation of Metallic Glassy Alloys of High Neutron Shielding Capability

**DOI:** 10.1038/srep36998

**Published:** 2016-11-16

**Authors:** J. C. Khong, D. Daisenberger, G. Burca, W. Kockelmann, A. S. Tremsin, J. Mi

**Affiliations:** 1 School of Engineering & Computer Science, University of Hull, Hull, HU6 7RX, East Yorkshire, UK; 2 Diamond Light Source, Oxfordshire, OX11 0DE, UK; 3 ISIS Neutron and Muon Source, Rutherford Appleton Laboratory, Oxfordshire, OX11 0QX, UK; 4 Space Sciences Laboratory, University of California at Berkeley, Berkeley, CA 94720, USA

## Abstract

This paper reports the design, making and characterisation of a series of Fe-based bulk metallic glass alloys with the aim of achieving the combined properties of high neutron absorption capability and sufficient glass forming ability. Synchrotron X-ray diffraction and pair distribution function methods were used to characterise the crystalline or amorphous states of the samples. Neutron transmission and macroscopic attenuation coefficients of the designed alloys were measured using energy resolved neutron imaging method and the very recently developed microchannel plate detector. The study found that the newly designed alloy (Fe_48_Cr_15_Mo_14_C_15_B_6_Gd_2_ with a glass forming ability of Ø5.8 mm) has the highest neutron absorption capability among all Fe-based bulk metallic glasses so far reported. It is a promising material for neutron shielding applications.

Since the 1980s, researchers in Inoue’s group in Tohoku University and Johnson’s group in California Institute of Technology have discovered a series of La-, Mg-, Zr-, Pd-, Fe-, Cu-, and Ti-based multicomponent alloys with large undercooling and low critical cooling rates (1–100 K/s) for glass forming^1^. All these alloys can be cast into bulk ingots or cylindrical bars with diameters in a range of a few millimetres to a few centimetres and were called bulk metallic glasses (BMGs)^1^,^2^,^3^. Most of the BMG alloys developed so far have high ultimate tensile strength^3^, high elastic limit^4^, and exceptionally high corrosion^5^ and wear resistance^6^ properties compared to their crystalline counterparts with the similar compositions. However, most of the alloys have very low ductility, typically <2% in compressive, and <1% in tensile load conditions^1^. Hence, structural engineering applications of BMGs have stalled because of the low ductility^4^. Recently, many attempts have been made to develop some of the BMGs into coating materials to make use of their exceptional corrosion and wear resistance capabilities. For example, Zhang, *et al.*^7^, Basu, *et al.*^8^ and Singh, *et al.*^9^ investigated the possibility of making Fe-based amorphous coatings using high velocity oxygen fuel (HVOF)^7^, laser selective deposition^8^ and spark plasma sintering^9^ methods. One particular promising Fe-based alloy is Fe_48_Cr_15_Mo_14_C_15_B_6_Y_2_ (named as Fe-B_6_Y_2_ hereafter)^3^. It has a glass forming ability of ~9 mm and can be manufactured into amorphous coatings of millimetre thickness onto different metal matrix materials. Blink, *et al.*^10^ at the Lawrence Livermore National Laboratory, USA investigated systemically a number of Fe-based BMG alloys with the aim of using them as coating materials for containers to store spent nuclear fuel^10^. They designed a new Fe-based alloy called SAM2X5 (Fe_49.7_Cr_17.7_Mo_7.4_W_1.6_Mn_1.9_Si_2.4_B_15.2_C_3.8_) and used HVOF method to spray successfully amorphous coatings on top of prototype nuclear waste containers^10^. The SAM2X5 amorphous coating not only provides very high corrosion resistance (14.3 to 15.9 μm/year in 90 °C seawater, and 26.1 to 29.7 μm/year in 90 °C NaCl solution), but also a certain level ionised radiation (especially neutrons) shielding capability^10^ for the nuclear waste containing long half-life radioactive elements or isotopes, for example, Cm-244 (18.1 years), Pu-238 (87.74 years), Pu-240 (6560 years), and Am-241 (433.6 years)^11^.

At the end of 2015, there are 441 nuclear power reactors in operation globally^12^, generating a total electricity of 382.9 GW(e). Thirty countries currently use nuclear power and about the same number are considering, planning or actively working to include it in their energy mix; and 68 new reactors are currently under construction^12^.

The US nuclear industry estimated that the total amount of commercial spent fuel was 71,775 metric tons at the end of 2013, including 22,233 metric tons in dry storage and other separate storage facilities^13^. It increases by an average of 2,150 metric tons annually for the entire US nuclear power industry. A long-term solution for high level and long lived radioactive waste is to store them in geologically stable repositories for natural decay^14^.

Hence, for containers to store the spent nuclear wastes, their materials must be able to withstand simultaneously the corrosion and oxidation caused by the humid and/or corrosive storage environment for very long time, and also have as much as possible neutron shield capability.

Currently, the materials that are often selected for making nuclear waste containers are copper, iron, stainless steels, titanium alloys and nickel-based alloys^15^. These materials have low neutron shielding capability, and coatings on top of those materials are needed to contain neutron radiation. The SAM2X5 was reported to have a neutron macroscopic attenuation coefficient (MAC) of 7.14 cm^−1^ at the thermal neutron energy (25.3 meV), which is more than 3 times higher than that of the borated stainless steel (2.28 cm^−1^) and twice as good as that of the Gd doped nickel-based alloy, C-4^10^ (Ni-Cr-Mo-Gd, 3.84 cm^−1^).

At present, the SAM2X5 is the best Fe-based amorphous alloys in terms of the combined capability of glass forming, neutron shielding and corrosion resistance reported in open literatures. Although extensive studies have been carried out to search for and/or design materials with combined very high hardness, high corrosion resistance together with high radiation shielding capability, solutions have not been found so far. In this paper, we report our recent research on designing a series of new Fe-based amorphous alloys with the aim of achieving the highest possible neutron shielding capability and sufficient glass forming capability.

## Design and making of the alloys

We designed and made a total of 15 Fe-based alloys with different compositions in the past 4 years through 3 rounds of progressive improvement in alloy compositions, glass forming ability and neutron shielding capability. The details can be found in Chapter 6 of Khong’s PhD thesis^16^. Due to the page limit, we just chose four typical alloys designed in this research (the chemical compositions are listed in Table 1) to describe our alloy development strategy and the important results. We used synchrotron X-ray diffraction (SXRD) to study the transition from crystalline to amorphous states for the alloys made under different cooling rates. The neutron shielding properties of these alloys were measured using the energy resolved neutron imaging technique available at the UK spallation neutron and muon source (ISIS, the Rutherford Appleton Laboratory, UK).

**Figure 1:**
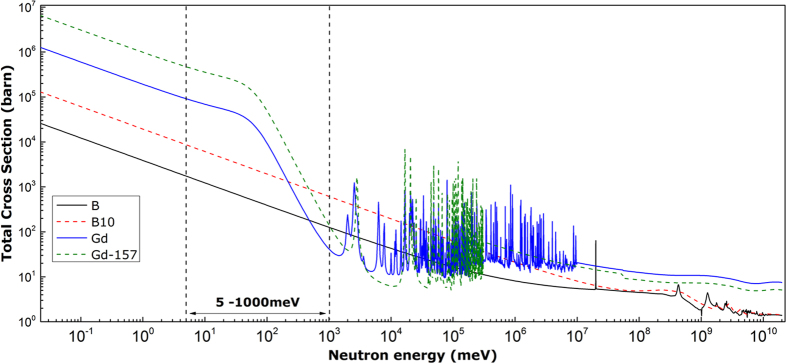
Total neutron cross section as a function of neutron energy for Gd-157 and B-10 isotopes^20^, and the calculated cross sections for the naturally occurring Gd that consists of 6 stable isotopes, and naturally occurring B that has two isotopes^21^. In the field of nuclear reactor physics^22^, neutrons can be roughly divided into three groups based on their energies: (1) thermal neutrons (25 meV–1 eV), (2) resonance neutrons (1 eV–1 keV), and (3) fast neutrons (1 keV–10 MeV).

For the alloys shown in Table 1, Fe-B_6_Y_2_ is the Fe_48_Cr_15_Mo_14_C_15_B_6_Y_2_ alloy reported by Ponnambalam, *at el*^3^, and was used here as the baseline to benchmark the glass forming ability and neutron transmission of the other four alloys. Fe is chosen as the matrix element because of its abundant resource and low cost. Cr and Mo are the major elements for contributing the corrosion and wear resistance properties for Fe based BMGs^17^,^18^. C contributes the hardness of the system. More importantly, C and B atoms have smaller radii and form the metalloids required for high glass-forming ability^19^. For enhancing neutron absorption, Gd was chosen because it has the highest total neutron cross section among all natural metal elements^20^,^21^. Figure 1 shows that, at the neutron energies below ~50 meV, the neutron cross section of Gd is nearly two orders of magnitude higher than that of B. Above ~50 meV, the neutron cross section of Gd begin to fall off but is still higher than B. However, in the energy range from ~500 meV to ~300 eV, i.e. within the resonance neutron energy range (1 eV–1 keV)^22^, the total cross section of Gd is lower than that of B except at the resonance peak energies. Therefore B can compensate the decrease of the total cross section of Gd in this energy range. In the fast neutron energy region (1 keV–10 MeV)^22^, although the total cross sections of both elements reduce significantly, Gd is still about 5–6 times higher than that of B^20^,^21^. In the designed alloys, Gd and B are therefore the most important elements that provide the capability of shielding neutrons over a wider energy range. The ultimate goal of this study is to design and tailor the concentrations of Gd, and B, and other major elements in the alloys to achieve the maximum neutron shielding capacity with highest possible glass forming ability. The Methods section describes in details how the alloys were made and analysed using synchrotron X-ray diffraction and neutron imaging methods.

## Results and Discussion

### Designed alloys and their glass forming ability

**Figure 2:**
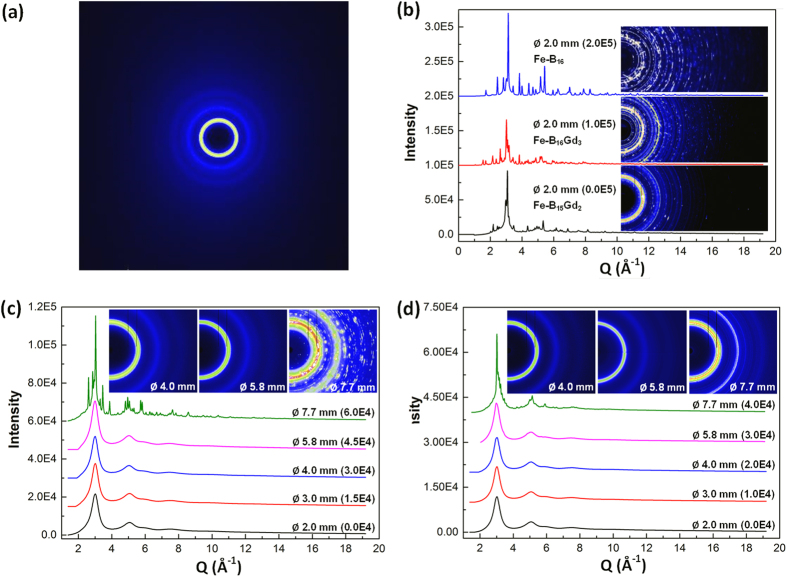
The selected 2D SXRD patterns acquired from the 2.5 ± 0.1 mm thick disk-shaped samples cut off from the different diameter sections of the as-cast step bars, and the corresponding integrated 1D profiles. (**a**) The 2D diffraction pattern acquired from the Fe-B_6_Gd_2_ alloy, (**b**) the Ø2 mm sections from the Fe-B_16_, Fe-B_16_Gd_3_, and Fe-B_15_Gd_2_ alloys, (**c**) different diameter sections from Fe-B_6_Gd_2_ alloy, and (**d**) different diameter sections from Fe-B_6_Y_2_ alloy.

Figure 2 shows the acquired 2D diffraction patterns (only a bit more than a half of the whole pattern is shown for those showed in Fig. 2b,c) and the corresponding integrated 1D profiles (stacked together with an equal distance of 1 × 10^5^ in Y direction) for the Ø2 mm sections of the Fe-B_16_, Fe-B_16_Gd_3_ and Fe-B_15_Gd_2_ alloy samples.

Compared to the lab-based X-ray diffraction technique used in characterising the SAM2X5 coatings^10^, mnochromatic synchrotron X-ray is much more sensitive in picking up nano crystal^23^ formed in the cast bars. The pair distribution function (PDF) derived based on the diffraction dataset is an ideal technique to study the short (<5 Å) and medium (5 Å~20 Å) range atomic structures^24^, especially for metallic glasses^25^. While long atomic range often refers an atomic distance of >20 Å^26^.

The empirical rules proposed by Inoue^27^ are used mainly in designing the alloys. They are based on (1) multiple elements, (2) significant atomic size mismatch >12% and (3) negative heats of mixing among the three main elements in the alloys. The designed alloys consist of elements of large atomic radius [Gd (1.74 Å) and Y(1.80 Å)], medium [Fe (1.28 Å), Cr (1.30 Å), Mn (1.32 Å), Mo (1.39 Å) and Nb (1.46 Å)], and small [C (0.77 Å), B (0.78 Å) and Si (1.02 Å)] atoms^28^. B, C, and Si are also metalloid elements for improving glass forming ability^29^. Fe-B_16_ was designed based on the composition of SAM2X5^10^ but replacing the very high melting point elements Mo (2896 K) and W (3695 K) with Nb (2750 K). The aim is to lower the melting temperature of the alloy, and at the same time to retain the glass forming ability. Unfortunately, the crystalline peaks in the integrated 1D profile and the Bragg spots in the 2D pattern (especially the needle-shaped features present in the 2D diffraction pattern) indicate that, for this particular composition, crystal structures form with possible change of orientation during the solidification process. Similar needle-shaped features were reported in the *in situ* study of TiAl during the transformation from α to γ phases using SXRD^30^. In our study, the research focus is on identifying the critical diameters of the as-cast step bars where transitions from crystalline to full amorphous state occur, hence the detailed structures of those crystalline phases formed in the non-amorphous diameter sections were not characterised.

Fe-B_16_Gd_3_ was designed based on Fe-B_16_ by replacing C_4_ with Gd_3_ and removing Mn completely. The aim is to have the highest atomic percentage of (B + Gd), i.e. 19 at.%, and also to study the possibility of using B without C to retain the glass forming capability.

The 1D profile shows that, compared with that of Fe-B_16_, the crystalline peaks are less intense, and the insert 2D pattern shows a faint halo ring and therefore suggests that the amorphous nature has improved. The result shows that the adding of 3 at.% Gd gives an apparent benefit in improving the glass forming capability.

Encouraged by the results from Fe-B_16_ and Fe-B_16_Gd_3_, we designed alloy Fe-B_15_Gd_2_. The diffraction pattern obtained from this alloy (Fig. 2b) shows even less crystalline features than that for Fe-B_16_Gd_3_. The 1^st^ and 2^nd^ amorphous halos are now visible in the 2D pattern and faint crystalline features are present in the integrated 1D profile. The results from Fe-B_15_Gd_2_ suggest that, with a further tuning of composition, it should be possible to design an alloy with more stable amorphous state. By reference to the Fe-B_6_Y_2_ alloy reported by Ponnambalam *et al.*^3^ and the recent work by Lavorato *et al.*^31^, it seems that the key for the fine tuning of the alloy composition is to tailor the ratio of C and B. Based on this judgment, the alloy Fe-B_6_Gd_2_ was made by replacing Y_2_ with Gd_2_ and retaining the ratio of C:B = 15:6.

Figure 2c shows the integrated 1D profiles (stacked with an equal distance of 1.5 × 10^4^ in Y direction) for the Ø2, 3, 4, 5.8 and 7.7 mm sections and the selected (corresponding) 2D diffraction patterns for the alloy Fe-B_6_Gd_2_. Clearly, for the sections of diameter less than or equal to Ø5.8 mm, a full amorphous state is achieved. Crystalline peaks only appear in the Ø7.7 mm diameter section, indicating that this alloy has a glass forming ability of at least ~Ø5.8 mm. We also studied the benchmark alloy, Fe-B_6_Y_2_, and the results are showed in Fig. 2d. Similarly to Fe-B_6_Gd_2_, a full amorphous state is retained up to Ø5.8 mm section. In the Ø7.7 mm section, minor crystalline peaks appear in the integrated 1D profile as sharp ring patterns appear in the corresponding 2D pattern. Based on the SXRD studies, we conclude that the alloy Fe-B_6_Gd_2_ has a similar glass forming ability as the widely studied Fe-based metallic glass alloy, Fe-B_6_Y_2_.

**Figure 3:**
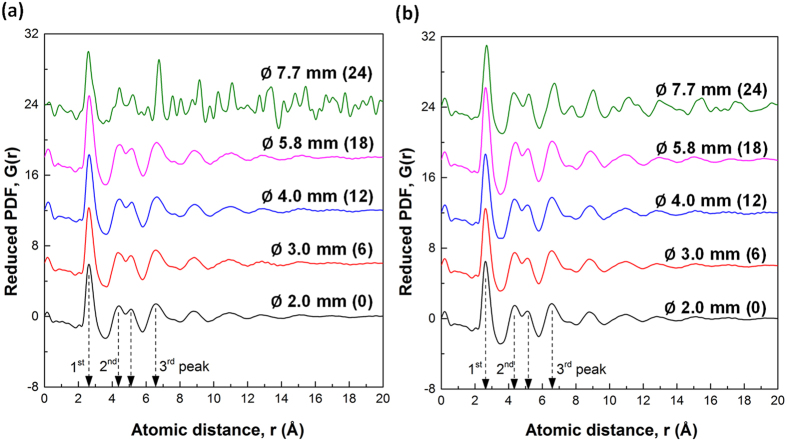
Reduced pair distribution function, G(r), calculated from the 1D SXRD profiles for different diameter sections of the step-bars of (**a**) Fe-B_6_Gd_2_ and (**b**) Fe-B_6_Y_2_ alloys.

To further characterise the amorphous state of the different diameter sections of the as-cast bars of the Fe-B_6_Gd_2_ and Fe-B_6_Y_2_ alloys, the pair distribution function (PDF) was used, and the results are showed in Fig. 3a and Fig. 3b for the different diameter sections (stacked with an equal distance of 6 in Y direction).

For Fe-B_6_Gd_2_ (Fig. 3a), all G(r) profiles are very similar until Ø5.8 mm, which all show the typically broad and diffuse peaks (the 1^st^, 2^nd^ and 3^rd^ peaks as marked on Fig. 3a) in the short and medium atomic ranges. However, at Ø7.7 mm, the three peaks become sharper, especially for the 3^rd^ peak. In addition, a series of small sharp peaks appear beyond the 3^rd^ peaks in the medium atomic range region. Actually, the corresponding 2D/1D diffraction patterns for the Ø7.7 mm section (Fig. 2c) clearly show that crystalline phases occur at Ø7.7 mm. We did not conduct any further analyses on those crystalline phases, because it is out of the scope of this study, and does not affect the conclusion of the research reported in this paper.

As for Fe-B_6_Y_2_ (Fig. 3b), it is very interesting to find that, for the diameter sections until Ø5.8 mm, the PDFs are almost identical to the corresponding PDFs showed in Fig. 3a. At Ø7.7 mm, the features of the first 3 peaks remain quite similar to those of the Ø5.8 mm case in terms of the peak position and the intensities. Beyond the 3^rd^ peak (>~7.6 Å), a series of small peaks appear in a repeated manner, suggesting crystalline phases appear at this diameter section, but apparently less intense than the Ø7.7 mm case showed in Fig. 3a. Both Y and Gd have 3 valence electrons with very similar ionization energy (600 kJ/mol for Y, and 593.4 kJ/mol for Gd), and similar atomic radius (1.80 Å for Y, and 1.74 Å for Gd). Compared Fig. 3a with 3b, it is interesting to find that the swap of Y with Gd resulted in almost identical atomic structures in the short and medium atomic distance regions, i.e. the same structures for the 1^st^, 2^nd^ and 3^rd^ atomic shells for the two alloys. Such identical structural information can only be obtained by using the PDF analyses on the data acquired from SXRD, which is impossible when lab-based XRD was used as in the work of Lavorato, *et al.*^31^ and that of Blink, *et al.*^10^ where much uncertainty remained on whether or not the cast plates^31^ and coatings^10^ made by them contained nanocrystalline phases. The combined SXRD and PDF analyses conclude that amorphous states can be achieved for both Fe-B_6_Gd_2_ and Fe-B_6_Y_2_ alloys up to Ø5.8 mm. The similar glass forming ability of Fe-B_6_Gd_2_ as that of Fe-B_6_Y_2_ indicates that Fe-B_6_Gd_2_ is a promising material for making amorphous coatings^32^.

### Neutron transmission

We used the pulse neutron beam produced by the spallation neutron source at ISIS, Rutherford Appleton Laboratory, UK to study the neutron absorption capabilities of the designed alloys. The details of sample preparation and experimental set up are described in the Methods section as well.

**Figure 4:**
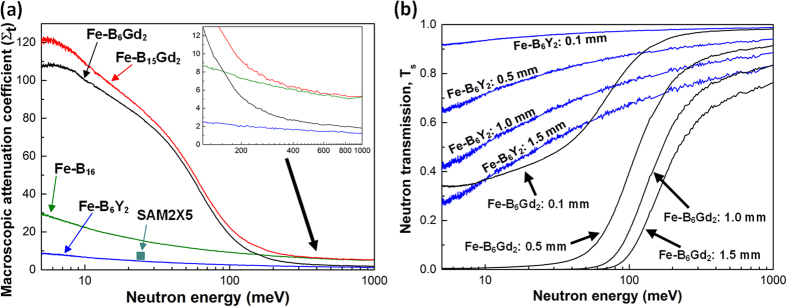
(**a**) The averaged MAC for the four alloys calculated based on the measurement of neutron transmission for each case in the range of 5–1000 meV neutron energy. The insert shows an enlarged area for the MAC from 200 meV above. (**b**) The calculated neutron transmission for the Fe-B_6_Y_2_ and Fe-B_6_Gd_2_ alloys at different thicknesses.

Figure 4 shows the averaged MAC and neutron transmission for the four alloys. The MAC of SAM2X5 at 25.3 meV is also showed on Fig. 4a (the green square).

Clearly, Fe-B_6_Y_2_ has the lowest MAC in this neutron energy range followed by Fe-B_16_. Actually, in both Fe-B_6_Y_2_ and Fe-B_16_, the neutron absorption contributions are mainly due to B, which has a total neutron cross section^21^ of 767 barn at 25.3 meV, and significantly higher than that of Cr (3.05 barn), Nb (1.15 barn), Mn (13.3 barn), Si (0.171 barn), C (0.0035 barn), Mo (2.48 barn), and Y (1.28 barn). Whereas, in addition to B, both Fe-B_6_Gd_2_ and Fe-B_15_Gd_2_ alloys contain Gd (49700 barn at 25.3 meV). In theory, Gd should provide approximately 2 orders of magnitude higher in neutron absorption than that of B in the energy range below ~50 meV. In this study, the neutron transmission measurement, and the calculated MAC in Fig. 4a give more systematic data for us to evaluate the designed alloys’ performance in terms of neutron absorption versus neutron energy. In the neutron energy of >200 meV, the four alloys actually show approximately the similar MAC trends in Fig. 4a. It is interesting to see that Fe-B_15_Gd_2_ has almost the same profile as that of Fe-B_16_ in the neutron energy of >400 meV, because the two alloys just have 1 at.% difference in B, while the 2 at.% Gd added into Fe-B_15_Gd_2_ does not enhance the neutron absorption capability anymore above 400 meV. This is simply because the drop of the neutron total cross section of Gd in that energy range as showed in Fig. 1. The insert figure in Fig. 4a also shows that the MAC of Fe-B_16_ is higher than that of Fe-B_6_Gd_2_ when neutron energy is >180 meV, indicating that B is taking over Gd in that energy range to provide the neutron absorption capability. Hence, both Gd and B are the essential alloy elements for designing alloys with high neutron absorption capability in a wider neutron energy range.

Fe-B_15_Gd_2_ is slightly better than Fe-B_6_Gd_2_ with the addition of the B component. However, the SXRD data show that Fe-B_15_Gd_2_ is a crystalline alloy, which will most likely to significantly reduce the corrosion resistance^33^. While Fe-B_6_Gd_2_ has a glass forming ability of Ø 5.8 mm, and the glass nature of the alloy allows a much more homogeneous distribution of Gd and B elements in the glassy matrix. Therefore at microlevel, the effect of neutron absorption will be much more homogeneous.

Figure 4b shows the effectiveness of Fe-B_6_Gd_2_ alloy for absorbing neutrons if used as a coating material with the thicknesses of 0.1, 0.5, 1.0 and 1.5 mm, and the comparisons with the benchmark Fe-B_6_Y_2_ alloy. At approximately below 100 meV, Fe-B_6_Gd_2_ alloy materials are generally performed much better than those of Fe-B_6_Y_2_ alloy materials. From engineering point of view, only 1/4 or 1/5 of the thickness of Fe-B_6_Gd_2_ alloy materials would achieve the similar neutron absorption capability as those of Fe-B_6_Y_2_ alloy materials. In the energy range of 10–50 meV, the 0.1 mm thick Fe-B_6_Gd_2_ alloy has a better neutron absorption capability than the 1.5 mm thick Fe-B_6_Y_2_ alloy material. So far, Fe-B_6_Gd_2_ is an amorphous alloy (with Ø 5.8 mm glass forming ability) with the highest neutron shielding capabilities. Currently, long-term corrosion resistance tests and neutron radiation induced crystallisation experiments for the designed alloy are underway to evaluate the long-term stability of the designed alloys.

## Conclusion

A systematic study on designing and developing new Fe-based amorphous alloys with high neutron absorption capability has been carried out. The transitions from crystalline to amorphous states for those alloys were characterised using synchrotron X-ray diffraction and pair distribution function methods. It was found that the alloy with the composition of Fe_48_Cr_15_Mo_14_C_15_B_6_Gd_2_ has the glass forming ability of at least Ø5.8 mm, similar to that of the widely studied bulk metallic glass alloy, Fe_48_Cr_15_Mo_14_C_15_B_6_Y_2._ Neutron transmission measurement confirms that Fe_48_Cr_15_Mo_14_C_15_B_6_Gd_2_ is the bulk metallic glass alloy with the highest neutron absorption capability reported so far.

## Methods

### Alloys and sample preparation

**Figure 5:**
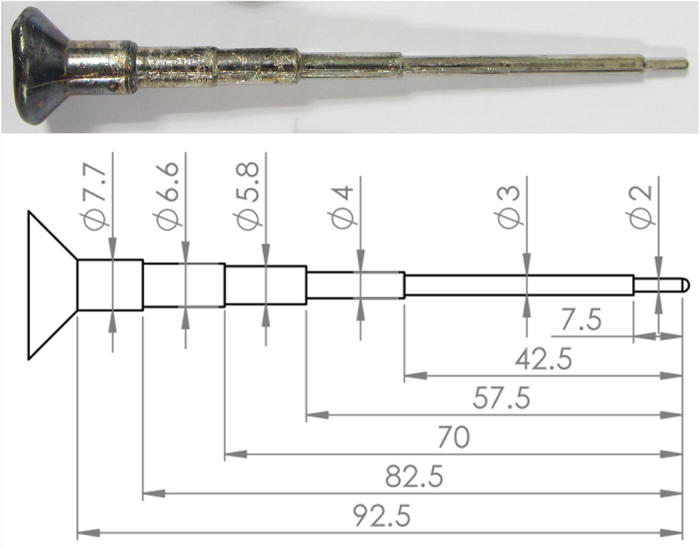
A photo and a drawing, showing the dimension of the as-cast step bar.

The designed alloy compositions are listed in Table 1. For each alloy, elements with purity of >99.9% were melted using an arc melting furnace in a Ti-gettered argon atmosphere^34^, and then cast into a copper mould to form step bars with different diameters as shown in Fig. 5.

For each SXRD measurement showed in Fig. 2, the samples were prepared using the following procedure: firstly, a 3 mm thick disk-shaped sample was cut off from the middle part of the different diameter sections of the as-cast step bar using a precision diamond wheel saw (Buehler IOSMET 1000). Then, the cut surfaces were ground using a P1200 SiC grinding paper to remove any irregular cutting groves or traces to ensure that the disk samples from different diameter sections have a consistent thickness of 2.5 ± 0.1 mm with smooth surfaces. In this way, for each sample, a consistent X-ray transmission thickness and gauge volume for X-ray scattering are maintained during the SXRD measurements.

For neutron transmission measurements, thin disk with a thickness of ~0.3 mm were used in the experiments. Thin disks with thicknesses of 0.5 mm was first machined off from the middle part of the different diameter sections of the step bars by electrical discharge machining. Both cut surfaces were ground to P2400 SiC paper, and then polished using 6, 3, and 1 μm diamond suspensions followed by 0.025 μm colloidal silica. The final thicknesses of the polished disks were measured using a micrometre screw gauge (±1 μm) and were taken into account in the neutron transmission calculations.

### Synchrotron X-ray diffraction experiment

The SXRD experiments were carried out at I15, The Extreme Condition Beamline, Diamond Light Source, UK. A monochromatic beam of 76 keV (*λ* = 0.1722 Å and Ø70 μm beamsize was used. A Perkin Elmer flat panel 1621EN 2D detector was used to acquire the 2D diffraction patterns. The sample-to-detector distance was set at 327.65 mm in order to achieve a high range of scattering vectors Q, (4*π sinθ*/*λ*), of ~20 Å^−1^. 20 s exposure times were used for each diffraction pattern acquisition. For each sample, five background patterns and five diffraction patterns were taken. The tilt, yaw, and sample-to-detector distance for all diffraction patterns were calibrated using the diffraction patterns obtained from a standard Si calibrant and the FIT2D software^35^.

### Pair distribution function analysis

Fit2D was used to obtain the integrated 1D profiles from the 2D patterns acquired from the background and the samples. The 1D profiles were then imported into software pdfgetX2^36^ for PDF analyses. The procedure is as follow:

1. The original sample 1D profiles, [*I*_*raw*_(*Q*)], were corrected for the normalization factor (*N*), X-ray polarization (*P*), and the calculated Compton scattering ;[*I*_*compton*_(*Q*)] from the sample alloy composition using Eq. 1.


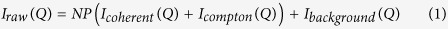


2. The correction was to extract the coherent intensity (*I*_*coherent*_(*Q*)) which was converted into the structure factor (*S(Q*)) using Eq. 2 37.


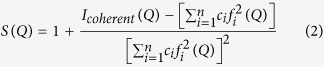


Where *c*_*i*_ and *f*_*i*_ are the atomic concentrations and atomic scattering factors^38^, respectively.

3. Finally, *S(Q*) was converted into the reduced pair distribution function (*G(r*)) by Fourier transform using the Eq. 3.


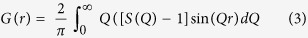


Where (*r*) is the atomic distance in Angstrom.

In theory, the calculation of *G(r*) needs Q to be available in the range of [0, ∞]. However, it is impossible to obtain an unlimited Q in real X-ray diffraction measurement, and the practical approach is to obtain a largest possible Q. Because Q = 4*π sinθ*/*λ*, a high energy (i.e. a shorter wavelength, *λ*) monochromatic X-ray beam is essential for obtaining a larger Q^16^,^34^.

### Time of flight neutron transmission measurement

**Figure 6:**
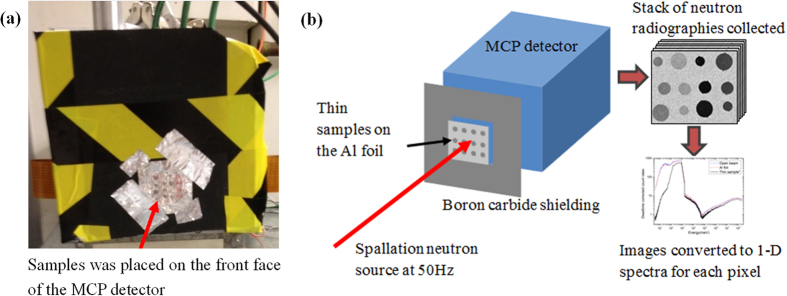
Time of flight neutron transmission experiment. (**a**) A photo, showing samples placed on the front face of the MCP detector where the actual detector plate is 12 mm behind the front face, (**b**) a schematic, showing the experimental setup, and neutron images acquired.

The neutron transmission measurements were carried out at the beamline ROTAX of the spallation neutron source, ISIS, UK. The spallation neutron source consists of a tungsten target and a cold 110 K methane neutron moderator at a pulse repetition rate of 50 Hz. A chopper system consisting of a ‘t-zero’ chopper and a disk chopper were used to filter out gamma radiation generated at the time of neutron production in the spallation target and to remove very slow neutrons below 5 meV from the incident beam, respectively. The flight path from the moderator to the detector was determined as 15.865 m by a calibration measurement, and then was used to calculate the TOF and neutron energies. The thin disk samples were carefully placed on a 30 × 30 mm^2^ pure Al foil with a thickness of 0.1 mm. The samples were held firmly and positioned using Al adhesive at the edge of a sample, leaving most of the area of the sample without any other obstacle for the incident neutron beam as shown in Fig. 6a.

A microchannel plate (MCP) detector with Timepix readout^39^ was used for the measurements. The detector^40^ has a detecting area of 28 × 28 mm^2^ and 512 × 512 total pixels with each pixel having a size of 55 × 55 μm^2^. The Al foil with the set of samples was then placed in front of the MCP detector and the sample-to-sensor distance was about 12 mm. In order to prevent any neutron damage to the readout electronics and reduce background neutron scattering, boron carbide shielding (the black sheet showed in Fig. 6a) was also placed around the detector area as illustrated in Fig. 6b.

**Figure 7:**
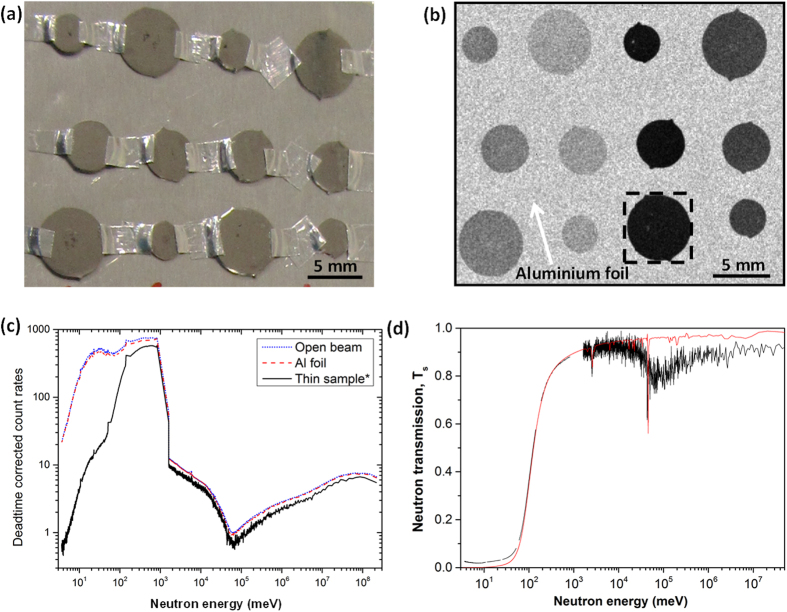
(**a**) The thin disk-shaped samples glued on the Al foil and the Time-of-flight neutron transmission measurement, (**b**) neutron transmission image at 80 meV, for the 12 thin disk samples mounted on the Al foil, (**c**) dead-time corrected TOF results for the open beam, the Al foil and the framed sample in Fig. 7b, (**d**) a corrected TOF neutron transmission profile (the black line) versus the calculated TOF neutron transmission for the framed sample (red line) showed in Fig. 7b.

Figure 7a shows the thin disk samples glued on the Al foil and Fig. 7b shows a typical Time-of-Flight (TOF) neutron transmission image (radiography) acquired at 80 meV for the thin disk samples. The count rates versus neutron energy recorded by the MCP in an ‘open beam’ condition for the Al foil and a selected sample after detector dead-time correction are shown in Fig. 7c. Open beam means images collected without the samples and the Al foil, which were used to normalize the difference in incident neutrons numbers at different energies. The irregular shape of the measured profiles are because of (1) a combination of the energy dependent beam flux profile on the beamline, (2) varying TOF bin widths for different sections of the covered time regime, and (3) at higher energies (shorter TOF values), the influence of the 50 Hz ‘t-zero’ chopper blade moving out of the beam. The drop of count rates at ~10^3^ meV is due to change of the bin-width from 20.48 μs to 0.48 μs.

For each pixel, the time of arrival of each detected neutron was registered and transferred into a histogram of 2676 time channels, i.e. a single measurement results in a stack of many time-of-flight radiographies (Fig. 6c). Therefore, to minimise the deadtime effects, the entire TOF range was separated into six regions separated by five 320 μs readout gaps and the acquired image sequence was further corrected for the detector deadtime using an overlap correction algorithm^40^. For all thin disk samples, transmission images with the energy range from 5 to 2 × 10^8^ meV were collected, but only the energy range from 5 to 1000 meV was analysed to investigate the neutron shielding capabilities for various energies of neutrons as Gd total cross section decreases rapidly in the epithermal neutron range.

The transmission *T*_*s*_ can then be calculated by dividing the sample images to the open beam images. However, in our case we chose a different procedure in order to remove the effect of the Al foil and improve the counting statistics. The analysis procedures are outlined below.

Firstly, the TOF neutron intensity was normalised by dividing the raw images data of the samples by the ‘open beam’ images. Secondly, the neutron TOF neutron transmission profile of a sample (with Al foil behind it) from a particular area of ~Ø10 pixels in diameter was extracted from each sample. Three areas were selected from each sample and their neutron transmission profiles were averaged, and taken as the transmission profiles for sample plus Al foil.

Secondly, the neutron attenuation generated from the Al foil needs to be corrected. The transmission profile of an empty area (with the Al foil but without sample) as shown in Fig. 7b was extracted from an area of the same size ~Ø10 pixels in diameter, and data from at least three different such locations were extracted and averaged, and taken as the Al only neutron transmission profile. Finally, the sample transmission was obtained by dividing the sample plus Al foil neutron transmission profile by the Al only neutron transmission profile.

This operation, of course, assumes that the incident neutron beam is spatially and spectrally homogeneous across the effective detector area of 28 × 28 mm^2^. Although the incident beam and the detector response are not uniform, both are normalized out by the open beam.

Figure 7d shows an example of the corrected TOF neutron transmission profile (the black line) versus the calculated TOF neutron transmission profile (red line) for the framed sample showed in Fig. 7b. The measured results agree with the calculation between 60 meV and 1000 meV. However, above 1000 meV, the neutron flux was significantly reduced because of the characteristics of the beamline, and the contribution of background signal becomes substantial. This leads to an increased noise level and increased errors in the measurement. Hence, the data analyses are limited to the measured data in the neutron energy from 5 to 1000 meV.

**Figure 8:**
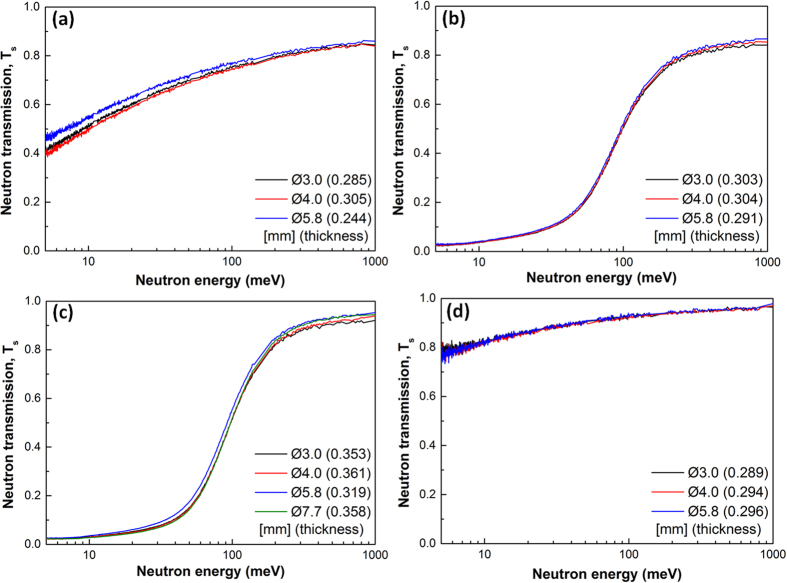
Measured neutron transmission in the neutron energy range of 5–1000 meV for different diameter disk samples with different thicknesses for four different alloys: (**a**) Fe-B_16_, (**b**) Fe-B_15_Gd_2_, (**c**) Fe-B_6_Gd_2_, and (**d**) Fe-B_6_Y_2_.

Figure 8 shows the measured neutron transmission profiles for the four alloy samples with different diameter sections and thicknesses. Because the neutron transmission measurements are dependent on the thickness of the sample, for easy comparison, the MAC (Σ_*t*_) for each alloy are calculated using the Lambert-Beer’s law in Eq. 4, and showed in Fig. 4a.


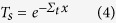


where *T*_*s*_ is the transmission coefficient, *x* is the thickness of the sample.

## Additional Information

**How to cite this article**: Khong, J. C. *et al.* Design and Characterisation of Metallic Glassy Alloys of High Neutron Shielding Capability. *Sci. Rep.* **6**, 36998; doi: 10.1038/srep36998 (2016).

**Publisher’s note:** Springer Nature remains neutral with regard to jurisdictional claims in published maps and institutional affiliations.

## Figures and Tables

**Table 1:** The compositions of the designed alloys and their glass forming ability.

Alloy	Nominal composition (atomic percent)	Glass forming ability (diameter, mm)
Fe-B_16_	Fe_52_Cr_19_Nb_4.5_Mn_2_Si_2.5_B_16_C_4_	<Ø 2
Fe-B_16_Gd_3_	Fe_50_Cr_22_Nb_4_Si_5_B_16_Gd_3_	<Ø 2
Fe-B_15_Gd_2_	Fe_48_Cr_15_Mo_14_C_6_B_15_Gd_2_	<Ø 2
Fe-B_6_Gd_2_	Fe_48_Cr_15_Mo_14_C_15_B_6_Gd_2_	Ø 5.8
Fe-B_6_Y_2_	Fe_48_Cr_15_Mo_14_C_15_B_6_Y_2_	Ø 5.8
